# Apontic regulates somatic stem cell numbers in Drosophila testes

**DOI:** 10.1186/s12861-016-0103-3

**Published:** 2016-03-18

**Authors:** Amanda J. Monahan, Michelle Starz-Gaiano

**Affiliations:** Department of Biological Sciences, University of Maryland Baltimore County, 1000 Hilltop Circle, Baltimore, MD 21250 USA; Present Address: Department of Medicine, Division of Infectious Disease, University of Massachusetts Medical School, Worcester, MA 01655 USA

**Keywords:** Stem cells, Apontic, Drosophila, Testes somatic stem cells, JAK/STAT signaling

## Abstract

**Background:**

Microenvironments called niches maintain resident stem cell populations by balancing self-renewal with differentiation, but the genetic regulation of this process is unclear. The niche of the Drosophila testis is well-characterized and genetically tractable, making it ideal for investigating the molecular regulation of stem cell biology. The JAK/STAT pathway, activated by signals from a niche component called the hub, maintains both germline and somatic stem cells.

**Results:**

This study investigated the molecular regulation of the JAK/STAT pathway in the stem cells of the Drosophila testis. We determined that the transcriptional regulator Apontic (Apt) acts in the somatic (cyst) stem cells (CySCs) to balance differentiation and maintenance. We found Apt functions as a negative feedback inhibitor of STAT activity, which enables cyst cell maturation. Simultaneous loss of the STAT regulators *apt* and *Socs36E,* or the *Stat92E*-targeting microRNA *miR-279,* expanded the somatic stem cell-like population*.*

**Conclusions:**

Genetic analysis revealed that a conserved genetic regulatory network limits JAK/STAT activity in the somatic stem cells of Drosophila testis. In these cells, we determined JAK/STAT signaling promotes *apt* expression. Then, Apt functions through Socs36E and *miR-279* to attenuate pathway activation, which is required for timely CySC differentiation. We propose that Apt acts as a core component of a STAT-regulatory circuit to prevent stem cell overpopulation and allow stem cell maturation.

**Electronic supplementary material:**

The online version of this article (doi:10.1186/s12861-016-0103-3) contains supplementary material, which is available to authorized users.

## Background

Adult stem cell maintenance is crucial for tissue homeostasis. Tissues with a high turnover rate, such as intestinal epithelium, sperm, and hematopoietic cells, rely on stem cells for constant replenishment of cellular populations [1, 2]. Further, damage to these and other tissue types require heightened proliferation of resident stem cells to facilitate tissue regeneration. However, this proliferation must be tightly regulated: stem cells must respond appropriately to growth demands while limiting the potential for tumorigenesis [3, 4]. Precise control of stem cell dynamics is governed by a specialized microenvironment called the niche [1, 5–9]. Short-range cues from the niche sustain the stem cell population, while allowing the production of differentiated daughters further away. Many niches are now known to regulate more than one stem cell type, and stem cells themselves or their direct progeny can sometimes function as a signaling component [5, 10].

The testis of *Drosophila melanogaster* provides a robust and genetically tractable system to study adult stem cells in their natural environment, and it has been well-characterized [11–15]. A cluster of 8–10 post-mitotic somatic cells comprises a major component of the niche, called the hub [16–18]. The hub supports germline stem cells (GSCs) and somatic cyst stem cells (CySCs). GSCs divide asymmetrically to self-renew and generate a gonialblast, which will give rise to mature sperm [13–15]. CySCs can divide to self-renew or generate cyst cells, which exit mitosis and, in pairs, encase each developing germ cell [15, 19, 20]. Mature cyst cells are required for GSC differentiation, which suggests CySCs can act as a signaling component of the niche [21–26].

The hub provides signals and structural organization to the niche, acting as a stem cell docking site. During development, hub cells undergo a change in gene expression, which includes the up-regulation of growth factors and cytokine-like molecules of the *unpaired* (*upd*) family [12, 16, 27, 28]. In Drosophila, Upd (also known as Outstretched/Os) is a secreted signaling molecule that activates the highly conserved Janus Kinase/Signal Transducer and Activator of Transcription (JAK/STAT) pathway [29, 30]. Upd released from the hub turns on JAK/STAT signaling in neighboring stem cells, which is required for them to stay undifferentiated [23, 24, 31–33].

JAK/STAT signaling is essential in a variety of organisms for normal development and stem cell maintenance [11, 12, 34, 35]. For example, mammalian hematopoeisis and inflammatory responses require cytokine signaling via JAK/STAT activation. The pathway must be tightly regulated as excessive STAT signaling correlates with tumorigenesis [36–39], and in Drosophila adult testis hyperactivation or loss of function of STAT (encoded by *Stat92E*) disrupts stem cell maintenance [23, 24, 31–33]. STAT activation promotes E-cadherin (E-cad)-directed adhesion of the GSCs to the hub, necessary for maintaining stem-cell properties [24]. In CySCs, STAT activity facilitates integrin-based adhesion to the hub and signals for self-renewal [31–33]. Two downstream targets of STAT, *zinc finger homeodomain-1* (*zfh-1*) [23] and *chronologically inappropriate morphogenesis* (*chinmo*) [21, 40] maintain CySC fate, in part by preventing the CySC-to-cyst cell transition. Ectopic STAT activation or Zfh-1 expression in the testis expands the CySC population, which prevents differentiation of both CySCs and GSCs [23, 24, 32, 33]. Since STAT activity must be finely tuned in stem cell populations to balance self-renewal and differentiation, it is important to understand how STAT regulation is genetically controlled.

The transcriptional regulator Apontic (Apt) is a key regulator of JAK/STAT signaling in the Drosophila ovary [41, 42]. During Drosophila oogenesis, a subset of germline-encasing, somatic epithelial (follicle) cells form an invasive cluster, called the border cells (recently reviewed in [43]). Border cell specification and migration require JAK/STAT signaling [44–46]. However, excessive pathway activation leads to additional invasive cells and a delay in cluster migration [41, 47, 48]. Apt restricts JAK/STAT activation in anterior follicle cells and limits migration to an optimal number of cells [41]. We, and others, have determined that Apt functions as a feedback inhibitor of STAT activity by regulating the expression of two direct STAT pathway inhibitors: *Suppressor of Cytokine Signaling at 36E* (*Socs36E*) and the STAT-targeting microRNA *miR-279* [47, 48]. This led us to investigate a role for Apt in other contexts.

Here, we report that Apt functions in the CySCs of adult testes to attenuate STAT signaling and limit stem cell numbers. As in ovaries, Apt expression in CySCs partially depends on STAT activity, and its feedback inhibition of STAT signaling functions through a regulatory network including *Socs36E* and *miR-279*. While other known STAT targets in CySCs promote self-renewal, Apt cell-autonomously permits timely stem cell differentiation. Our data demonstrate that Apt facilitates robust regulation of STAT activity to regulate stem cell numbers in Drosophila testes.

## Methods

### Fly stocks and husbandry

Flies were raised on standard cornmeal-molasses food; all crosses were performed at 25 °C. The following fly stocks were utilized: Canton S and *w*^*1118*^ (for wild type), *tubP-Gal80*^*ts*^ [49], *upd-Gal4* (expressed in hub [22]), *c587-Gal4* (expressed in CySCs and early cyst cells [50]), *Tj-Gal4* (expressed in hub, CySCs, and early cyst cells [51, 52]), *UAS-tdf/MKRS* (for over-expression of *apt; tdf* is an alternative name for *apt* [53]), protein trap line *PTT-GC apt*^*CC01186*^ [54, 55], *Stat92E*^*397*^*/TM3* (a null allele of *Stat92E*) [46], two independent null *miR-279* alleles (*miR-279*^Δ*1.2*^ and *miR-279*^Δ*1.9*^ [56]), *miR-279sponge* [48], *UAS-Hop*^*TUM-L*^*/CyO* [57], and *UAS-mCD8-GFP* [58]. The *apt* loss-of-function mutant alleles used were: *apt*^*KG05830*^ [41, 59], *apt*^*tdf-P*Δ*4*^*/CyO* [53], and *apt*^*167*^*/CyO* [60]. The TRiP collection provided: *UAS-aptRNAi* (TRiP.JF02134), *UAS-updRNAi* (TRiP.JF03149), and two *UAS*-*Stat92E-RNAi* lines (TRiP.JF01265 = *stat RNAi*^*31317*^ and TRiP.GL00437 = *stat RNAi*^*35600*^) [61, 62].

Inverse PCR was utilized to confirm and map the P-element insertion site of the protein trap *PTT-GC apt*^*CC01186*^. Genomic DNA was isolated from *apt*^*CC01186*^ flies. DNA was subsequently digested with *PvuII * (Fermentas) or *Msp1* (Fermentas) overnight at 37 °C. An overnight ligation reaction (T4 DNA Ligase – Thermo Scientific) was performed at 4 °C on the digested DNA to promote self-ligation of the fragments. Ligation products were amplified with Pry1 (5’ CCT TAG CAT GTC CGT GGG GTT TGA AT 3’) and Pry4 (5’ CAA TCA TAT CGC TGT CTC ACT CA 3’) primers at an annealing temperature of 55 °C. Purified PCR products were sequenced with the PEP1 (5’TAC GAC ACT CAG AAT ACT ATT C 3’) primer by Genewiz. Blastn (http://blast.ncbi.nlm.nih.gov/Blast.cgi) and Flybase (www.flybase.org) were utilized to analyze sequences.

To rescue the *aptRNAi* phenotype, *c587-Gal4; UAS-aptRNAi/CyO* flies were crossed to *UAS-tdf(apt)/MKRS* [53]; offspring and controls were incubated at 29 °C for 2 days prior to dissection. To generate *Socs36E* deficient flies *Socs36E*^*EY06665*^ and *Socs36E*^*178*^ were crossed to produce transheterozygotes [47]. To test for a *Socs36E, apt* genetic interaction, two independently derived stocks of the genotype *Socs36E*^*EY06665*^*, apt*^*167*^*/CyO* were crossed to *Socs36E*^*178*^. To create *Socs36E, apt* double mutants, two *Socs36E*^*EY06665*^*, apt*^*167*^*/CyO* lines were crossed with a single recombinant stock *Socs36E*^*178*^*, apt*^*KG05830*^ [47].

Flies bearing mutant alleles were kept at 25 °C for 0–2 days prior to dissection. Gal4 containing males were incubated at 29 °C for 2 days before dissection for effective RNAi expression. For genotypes in which *c587-Gal4* or *upd-Gal4* was combined with *tubP-Gal80*^*ts*^ and for the temperature matched controls, 0–2 day old experimental and temperature matched control males were shifted to 30 °C for 4 days. Age-and-genotype-matched control males were kept unshifted at 25 °C for 4 days. Males generated for experimental analysis were maintained at less than 20 males per vial and were transferred onto fresh food every 2–3 days until dissection.

### Testes dissections and immunofluorescence

Males were dissected in Schneider’s media containing 10 % Fetal Bovine Serum (FBS) and 0.3X Pen/Strep antibiotics (50 mg/mL, ThermoFisher). Testes were fixed for 10 min at room temperature (RT) in 4 % paraformaldehyde in PBX (PBS with 0.1 % Triton-X), washed at RT with PBX, and blocked for 1 h at RT (PBX with 2 % goat serum and 3 % bovine serum albumin (BSA)). Antibodies were diluted in block and incubated with testes overnight at 4 °C. Testes were washed with PBX prior to addition of Molecular Probes AlexaFluor secondary antibodies (488 nm and 568 nm), which were diluted at 1:200 in PBX and incubated overnight at 4 °C. DAPI was applied at 1:1000 (in PBX) for 10 min at RT; testes were washed with PBX, and stored in 50 % glycerol at 4 °C until mounted for imaging.

The following primary antibodies were utilized for analysis: rabbit anti-Apontic (1:500, provided by Dr. S. Hirose [63]), rabbit anti-STAT (1:100, provided by Dr. Denise Montell [64]), rabbit anti-Zfh-1 (1:5000; provided by Dr. Ruth Lehmann [65]), rabbit anti-Vasa (1:1000, provided by Dr. Ruth Lehmann [65]), guinea pig anti-Traffic jam (1:5000, provided by Dr. Dorthea Godt [66], mouse anti-GFP (1:1000, Molecular Probes), and mouse anti-BrdU conjugated with fluorophore Alexa 488 (1:40; Molecular Probes). The following antibodies were obtained from the Developmental Studies Hybridoma Bank, developed under the auspices of the NICHD, and maintained by the University of Iowa, Department of Biology, Iowa City, IA 52242: mouse anti-Fasciclin 3 (Fas3, 1:50, 7G10: Goodman, C. [67]); rat anti-DCad2 (for E-cadherin, 1:25, DCAD2: Uemura, T. [68]), rat anti-N-Cadherin (N-Cad, 1:25, DN-EX #8 Uemura, T. [69, 70]); mouse anti-Eyes Absent (1:100, EYA10H6: [71]).

Images were obtained with a Zeiss AxioImager Z.1 microscope equipped with AxioVision software and ApoTome structural interference system for optical sectioning or a Leica TCS 4D Scanning Confocal Light Microscope. Images of testes stained with anti-Zfh-1 (except Fig. 2a-b and Additional file 3: Figure S3A, C, E) are 3D reconstructions projected into 2D, generated by FIJI software [72]. All other images are a representative optical section of a Z-stack. Adobe Photoshop CS6 and FIJI software were utilized to process and format images.

### Analysis and quantification of GSCs and total Zfh-1-positive cell population

To determine the number of CySCs or GSCs in the testis, we used Zeiss AxioVision or Leica LAS software to generate Z-stacks of optical sections of the apex in 0.5–1.0 μm steps. To be considered a GSC, a single Vasa-positive cell had to be in direct contact with the hub. FIJI or Zeiss AxioVision software was utilized to step through the Z-stack to quantify all GSCs in a single testis. For Zfh-1 expression analysis, we counted all cells except hub cells that stained positively with a rabbit antibody directed against Zfh-1, either stepwise through a Z-stack with Zeiss AxioVision software or through generation of a 3D reconstruction of the testis with Zeiss AxioVision or PerkinElmer Volocity software. We capped Zfh-1+ cell counts at 100 per single testis, since if there were more cells they were very far from the niche. When testes were co-stained with anti-Zfh-1 and anti-Eya antibodies, Zeiss AxioVision and FIJI software were utilized to assess co-expression of markers.

### Quantification of relative expression levels via fluorescent analysis

To obtain relative levels of Apt or STAT protein expression, all dissections and antibody stainings for a single experiment were performed together on the same day. Images for each experiment were also acquired in a single day, with the same exposure times. For analysis, we used FIJI software to circle nuclei with the freeform selector, and then used the measure tool to obtain average pixel intensity of several stem cells per testis in both the anti-Apt or anti-STAT and DAPI positive channels [72]. For this study, we defined CySCs as the first tier of Zfh-1 or Tj-positive cells around the hub. DAPI was used as an internal control to obtain a relative level of Apt expression by generating an Apt/DAPI ratio. To acquire a normalized expression level of Apt relative to the control, we set the control to one and derived an Experimental/Control ratio to calculate a relative fold-change in our experimental genotype(s).

### Analysis of E-cadherin expression levels

To assess E-cad expression, testes from *w*^*1118*^ and *apt*^*KG05830*^ homozygous males were dissected and stained with antibodies on the same day. Images for each experiment were also acquired on a single day, with the same exposure times. Images were analyzed as single optical sections and as a stack of optical sections projected in the 2D by FIJI/Image J. Projections for both genotypes were constructed from a similar number of optical sections and represent a similar depth of tissue.

### In vivo BrdU labeling

Testes were labeled with BrdU (Invitrogen/Life Sciences B23151) as previously described [73]. Age-matched males of control and experimental genotypes were incubated for 2 days at 29 °C, for effective *RNAi* expression. During this incubation, males were starved for 4 h. Starved males were then fed 2.5 mM BrdU (diluted in PBS-apple juice to encourage eating, and 6 % green food coloring) for the final 20–24 h of the 29 °C incubation. Only males with green abdomens were dissected. The same protocol described above for dissections was then utilized.

### Statistical analysis

All statistical analyses on cell count and expression data were executed via a two-tailed t-test. Two-tailed Fisher’s Exact test (http://www.graphpad.com/quickcalcs/contingency1.cfm) were utilized for statistical analysis of phenotypic penetrance. Cell count data are displayed via non-parametric box and whisker plots. In these plots, the second (lower) and third (upper) quartile bars are separated by the median value. The diamond specifies the mean for each genotype. The upper whisker indicates the upper quartile through the maximum, while the lower whisker shows the minimum value observed through the first quartile. The standard deviation is provided (+/-) for all quantitative data not presented in graphs. For all statistical analysis, we maintained a significance requirement of at least *p* < 0.05.

## Results

### Apontic protein is enriched in the adult testis apex

**Fig. 1:**
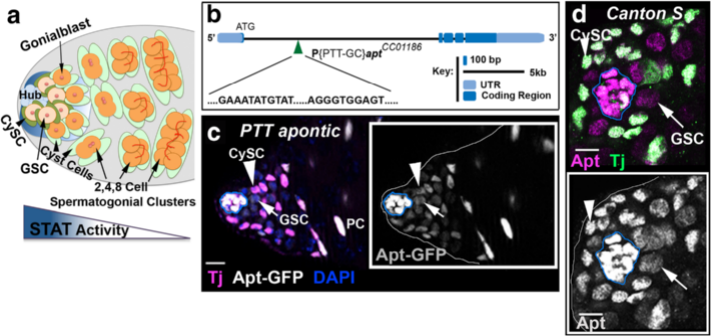
Apontic expression is enriched in the testis apex. **a** Schematic of the adult Drosophila testis apex. 8-10 germline stem cells (GSCs, tan) adhere to the hub (light blue circles) and divide asymmetrically to self-renew and generate a differentiated daughter, the gonialblast. Cell division with incomplete cytokinesis produces 2, 4, 8, and 16 (not drawn) cell-cluster spermatogonia. A pair of somatic cyst stem cells (CySCs, green) associates with each GSC, dividing asymmetrically to produce non-mitotic cyst cells (light green), which grow and encase the developing germ cell. Red indicates the fusome, which is a small sphere (dot) in GSCs and gonialblasts but is branched in spermatogonium. Blue gradient behind the hub and triangle below schematic represents STAT activity: the highest level of STAT signaling (dark blue) is found in cells adjacent to the hub, and it decreases distally (light blue to white). **b** Schematic of the *PTTapt* ^*CC01186*^ P-element insertion within the *apt RA* transcript (boxes represent exons, black line indicates introns). Green triangle illustrates P-element insertion site of the GFP protein trap (*PTT-apt*) at genome region 23,586,285, in which *gfp* can be spliced into the *apt* mRNA to generate a full-length Apt protein fused to GFP. **c-d** Single optical section through the testis apex of males of the specified genotypes, fluorescently stained with denoted antibodies. Arrows indicate germline cells, arrowheads CySCs, the hub perimeter is outlined in blue, and the testis tip is outlined in white. Scale bars = 10 μm. **c** *PTTapt* ^*CC01186*^ protein-trap line reveals Apt expression (Apt-GFP, white). Traffic Jam (Tj, magenta) co-localization indicates that Apt is expressed in the somatic lineage (hub, CySCs, and mature cyst cells), as well as in GSCs and gonialblasts (Tj-negative, Apt-GFP+). DAPI (blue) stains nuclei. PC - pigment cell. Inset displays Apt reporter expression alone. **d** A testis from a Canton S male stained with an antibody directed against Apt. Apt (magenta) is expressed in the soma (co-labeled by Tj expression, green) and GSCs, and at a lower level in gonialblasts, further from the hub. Inset shows Apt staining alone

At the apex of the testis two stem cell populations surround the dome-shaped hub: somatic cyst stem cells (CySCs) and germline stem cells (GSCs) (Fig. 1a and [13–15]). To examine Apt expression in the adult testis, we first used a protein trap for *apt* (*PTT-apt*^*CC01186*^), which is predicted to create a GFP tagged full-length protein through mRNA splicing [55]. We confirmed the location of the insertion to be downstream of at least one exon for all isoforms of *apt* (Fig. 1b). This reporter revealed enriched Apt-GFP expression in the hub and surrounding cells (Fig. 1c). By co-staining with an antibody directed against the somatic cell marker Traffic jam (Tj) [66], we confirmed that Apt-GFP is expressed in the nuclei of CySCs, mature cyst cells, and distal pigment cells [74], consistent with a previous report [75]. We also detected Apt-GFP expression in the GSCs. An antibody directed against Apt showed the same expression pattern in testes from wild - type males (Fig. 1d) and overlapping expression with Apt-GFP (Additional file 1: Figure S1).

### STAT activity promotes *apontic* expression in CySCs

**Fig. 2:**
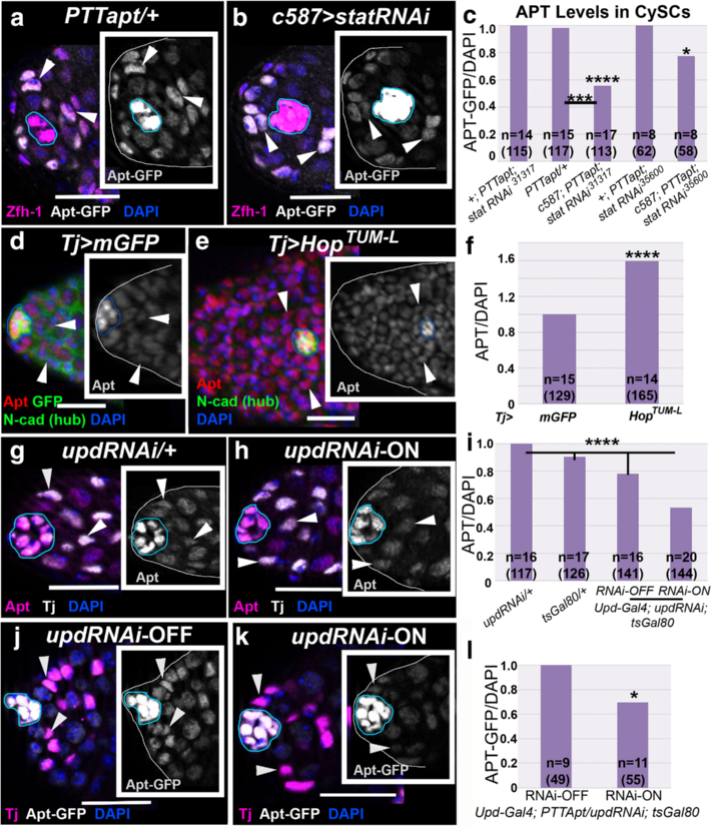
Apontic is a downstream target of STAT. **a-b** Optical sections of testes from males of the specified genotypes in a *PTTapt* ^*CC01186*^ genetic background, stained with antibodies directed against Zfh-1 (magenta) and GFP (white, to report Apt) and DAPI to label nuclei (blue). Insets display Apt-GFP expression alone. Arrowheads indicate CySCs. For all optical sections, a blue line outlines the hub perimeter, the first tier of Zfh-1+ or Tj+ cells around the hub were considered CySCs, and the scale bar = 20 μm. **a** Apt reporter expression in CySCs is wild type in the *PTTapt/*+ control, and is lower when *stat* is reduced in CySCs as in (**b**) *c587-Gal4; PTTapt; UAS-statRNAi* ^*TRiP.JF01265*^ (compare arrowheads). **c** Apt-GFP expression is significantly reduced in CySCs nuclei when *stat* is lowered in these cells, measured by pixel intensity relative to the no driver controls or *PTTapt* alone, normalized to DAPI (see Methods). Statistical analysis was performed between experimental and the no Gal4 driver controls, unless indicated by bar. P-values for graphs (**c, f, i, l**) are from two-tailed t-tests where *p<0.05; **p<0.01; ***p<0.005; ****p<0.0001. For graphs, “n” represents total testes analyzed; number of cells measured is in parentheses. **d-e** Single section images of testes, stained with antibodies specific for Apt (red) and NCad (green, hub) and GFP (green, (**d**)), counterstained with DAPI (blue). Expression of a constitutively active JAK mutant in the CySCs and early cyst cells via *Tj-Gal4* heightens nuclear Apt protein levels (**e**), relative to the GFP control (**d**). Insets show Apt expression. **f** Quantification of Apt, as in (**c**): Apt protein levels are significantly increased when JAK/STAT is activated in the soma. **g-h** Optical sections of testes stained with antibodies directed against Apt (magenta) and Tj (white) and counterstained with DAPI (blue). Insets display Apt alone. **g** Testis from a control male (*UAS-updRNAi*/+, no Gal4 driver, incubated at 30 °C) shows wild-type levels of Apt.  **h** Apt expression is decreased in CySCs when *upd *is reduced in the hub of adult males (*upd-Gal4; updRNAi; tsGal80-*RNAi-ON, 30 °C, restrictive temperature for tsGal80). **i** Apt is significantly reduced in CySCs when *upd* is disrupted in the adult hub, relative to controls. With the exception of *upd-Gal4; updRNAi; tsGal80*-RNAi-OFF flies, all adult males were incubated at 30 °C for 4 days prior to dissection. **j-k** Optical sections of testes from males bearing *upd-Gal4, updRNAi,* and *tub-tsGal80* (to regulate RNAi expression temporally) transgenes in a *PTTapt* genetic background, stained with antibodies, as in (**a-b**). Insets display Apt-GFP alone. **j** Testis from a control male (RNAi-OFF, 25 °C, permissive for tsGal80 repression) shows wild-type levels of Apt-GFP (white). **k** Apt (white) expression is decreased in CySCs when *upd* is reduced in the hub of adult males (RNAi-ON, same genotype as (**j**), 30 °C, restrictive temperature for tsGal80). **l** Apt-GFP is significantly reduced in CySCs when *upd* is disrupted in the adult hub, relative to control males

STAT signaling activates Apt expression in the ovary [41], so we examined this possibility in testis. Prior work determined that activated STAT binds to several sites in the *apt* gene regulatory region [41]. In addition, Apt is expressed in testis stem cells (Fig. 1c-d, and [75]), which exhibit high levels of STAT activity [11, 12]. To alter signaling levels and assay Apt expression, we utilized the tissue-specific Gal4/*UAS* system [76]. First, we reduced *Stat92E* specifically in CySCs and early cyst cells to assess cell-autonomous effects on Apt expression. Reduction of *Stat92E* with either of two RNAi lines, which we have previously verified [77], expressed via the *c587-Gal4* driver [22], significantly lowered Apt reporter expression in CySCs, the first tier of Zfh-1-positive (Zfh-1+) cells around the hub (Fig. 2a-c, see Methods). Conversely, constitutive activation of JAK in the somatic cell population (*Tj-Gal4/UAS-Hop*^*TUM-L*^) [57] resulted in heightened Apt protein levels in each cell (Fig. 2d-f).

To reduce STAT activity in stem cells non-autonomously, we reduced expression of the activator, Upd, by expressing a previously-verified RNAi line [78] in adults with the hub-specific Gal4 driver *upd-Gal4* [16, 79] and a temperature sensitive Gal4 repressor, Gal80 (tsGal80) [49]. The presence of tsGal80 allowed normal *upd* expression throughout development at 25 °C, including during hub formation and stem cell establishment in embryonic testes [16]. Adult males were shifted to 30 °C to deactivate tsGal80 and allow hub-specific expression of *UAS*-*updRNAi*. Again, examination of Apt protein by antibody staining (compare Fig. 2g and h and see i), or GFP expression in the *PTT-apt*^*CC01186*^ line (compare Fig. 2j and k, and see l), revealed a significantly lower level of Apt expression in CySCs (the first tier of Tj + cells around the hub) when *upd* was reduced (RNAi-ON), relative to temperature-matched controls (RNAi-OFF). The residual Apt expression in the *Stat92ERNAi* and *updRNAi* experiments indicates that other factors contribute to *apt* expression in the testis. However, considered with the prior DNA binding data [41], these results suggest that *apt* is a downstream target of STAT activity in CySCs of the testis.

### Apt limits the CySC/early cyst cell population and permits timely differentiation

**Fig. 3:**
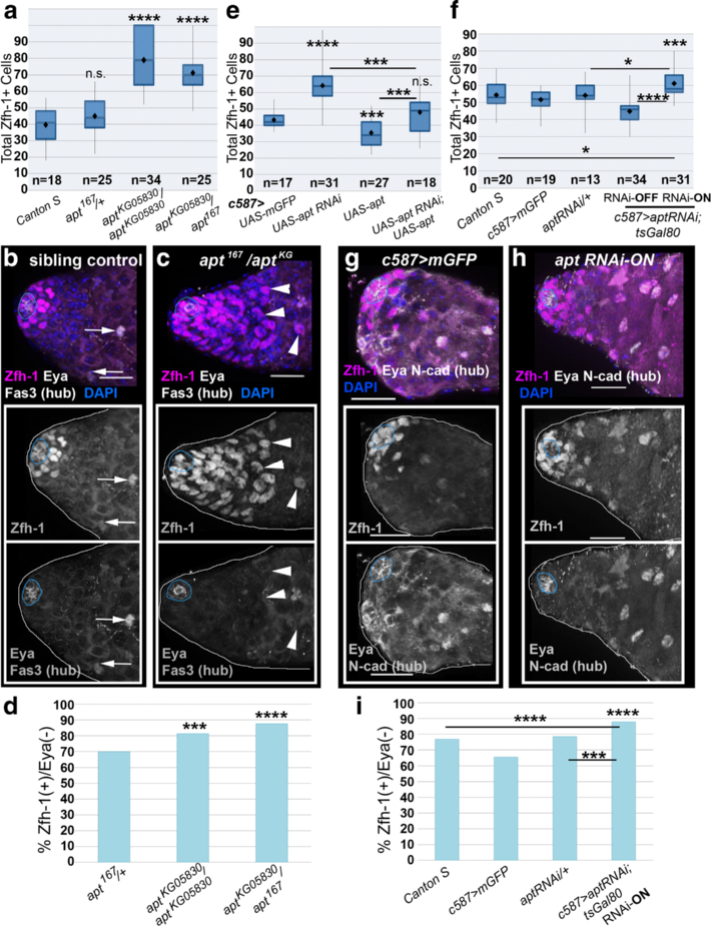
Apontic limits the Zfh-1+ population. **a** Total number of Zfh-1+ cells in adult testes from flies of the specified genotypes, represented by a box and whisker plot: diamond gives the mean, horizontal line indicates the median (see Methods). For a single testis, cell quantification was capped at 100, so graphs likely represent an underestimate. **b-c** Stacks of optical sections, projected into 2D, of testes stained with the following antibodies: Zfh-1 expression (magenta) marks CySCs and immediate daughters, Eyes absent (Eya, white) labels mature cyst cells, and Fas3 indicates the hub (white, blue outline). Insets display Zfh-1 or Eya/Fas3 staining alone. **b** In control testes (*apt* ^*167*^/+), wild-type numbers of Zfh-1+ cells are found proximal to the hub. Arrows indicate Zfh-1+/Eya+ distal somatic cells. **c** In testes from an *apt* ^*167*^ */apt* ^*KG05830*^ mutant male, the Zfh-1+/Eya- population is increased around and distal to the hub, unlike in wild type (arrowheads). **d** Total percent of Zfh-1+ cells that did not co-express the differentiation marker Eya in testes from the experiment in (**a**). **e-f** Total numbers of Zfh-1+ cells in adult testes from flies of the specified genotypes. Males from all genotypes tested in (**f**) (except *c587; aptRNAi; tsGal80* –RNAi-OFF) were incubated for 4 days at 30 °C prior to dissection. **g-h** Stacks of optical sections, projected into 2D, of testes from males incubated at 30 °C for 4 days stained with antibodies directed against Zfh-1 (magenta), Eya (white), and NCad (white). DAPI (blue) labels nuclei. Insets show Zfh-1 or Eya/NCad channels. **g** Control males display wild-type numbers and arrangements of the Zfh-1+ population. **h** A testis from a *c587; aptRNAi; tsGal80* male kept at the tsGal80 restrictive temperature, reducing *apt* in CySCs and early cyst cells, shows an expanded Zfh-1+ population (RNAi-ON). **i** Total percent of Zfh-1+ cells that did not co-express the differentiation marker Eya in testes of the indicated genotypes from experiment in (**f**). For (**a, e, f**), two-tailed t-tests measured significance of the difference compared to *apt* ^*167*^/+ (**a**) or *c587; mGFP* (**e, f**) unless otherwise indicated by a bar. For (**d, i**) two-tailed Fisher’s exact tests measured significance of the difference compared to *apt* ^*167*^/+ (**d**) or *mGFP* control (**i**), unless otherwise indicated by bar. For all statistical tests: **p<0.01, ***p<0.005, ****p<0.0001; n.s., not significant. “n” indicates number of testes scored. For all images scale bars = 20 μm

Given Apt’s expression pattern, we wanted to determine whether *apt* mutations affected stem cell maintenance. We analyzed testes in which *apt* was reduced using characterized mutant alleles: *apt*^*KG05830*^*,* a homozygous viable P-element insertion line [41, 59], and *apt*^*167*^, which contains a point mutation in the conserved DNA binding domain [41, 60]. The *apt*^*167*^ allele acts as a functional null and is homozygous lethal [60], but is viable *in trans* to the hypomorphic allele *apt*^*KG05830*^. To assess CySCs, we examined expression of Zfh-1, a STAT downstream target that is necessary and sufficient for CySC maintenance [23]. *apt*^*KG05830*^ homozygotes and *apt*^*KG05830*^*/apt*^*167*^ males both displayed a significant increase in the Zfh-1+ population near the hub compared to controls, suggesting the presence of additional CySCs or early cyst cells (Fig. 3a-c). Analysis of the Tj + population in testes from these males confirmed that the somatic population is expanded when *apt* is lost, relative to controls (Additional file 2: Figure S2A-B). To clarify the identity of these cells, we co-stained testes from *apt* mutants with antibodies directed against Zfh-1 as well as Eyes Absent (Eya), a marker for mature cyst cells that is not expressed in CySCs or early cyst cells [23, 80]. Significantly more Zfh-1+ cells are Eya-negative in *apt* mutants, relative to controls (Fig. 3b-d). Furthermore, some Zfh-1+, Eya(-) cells were found to be distal from the hub, which was not often observed in wild - type testes (arrowheads in Fig. 3c, compare with arrows in b). This spatial pattern of Zfh-1 and Eya expression in *apt* mutants indicates a delay in cyst cell maturation.

To determine if *apt* loss of function caused the cyst cell phenotype cell-autonomously, we reduced *apt* specifically in CySCs and early cyst cells via *c587-Gal4* and a *UAS*-controlled RNAi line targeting *apt.* Analysis in ovaries confirmed that expression of *aptRNAi* in follicle cells phenocopied *apt* loss of function mutants (Additional file 2: Figure S2C-D and [41]). Consistent with *apt* mutant alleles, tissue-specific reduction of *apt* showed a significantly expanded Zfh-1+ population (Fig. 3e, Additional file 3: Figure S3A-D). We observed similar results using the *Tj-Gal4* line, which drives expression in the hub, CySCs, and early cyst cells (Additional file 3: Figure S3H, [52]). Re-introduction of full-length *apt* via *c587-Gal4* confirmed that the observed phenotype was due to loss of *apt*, as it restored wild-type number and organization of Zfh-1+ cells (Fig. 3e and Additional file 3: Figure S3E-F). In contrast, over-expression of *apt* in CySCs in a wild-type background significantly reduced the number of Zfh-1+ cells, which often led to complete localization of the Zfh-1+ population next to the hub, and/or a significant decrease in the percentage of Zfh-1+, Eya(-) cells, relative to controls (Fig. 3e, Additional file 3: Figure S3G-H). Next, to prevent possible phenotypes caused by early loss of *apt*, we combined tsGal80 with *c587*-*Gal4* and *aptRNAi*. We found a significant expansion of the Zfh-1+ population when *apt* was reduced in adults, relative to the controls (Fig. 3f-h). Again, co-expression of Zfh-1 and the differentiation marker Eya was less prevalent when *apt* was reduced in adults, relative to temperature-matched controls (Fig. 3i).

**Fig. 4:**
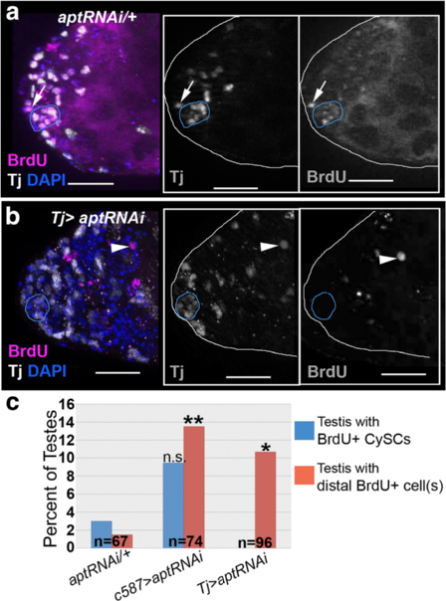
Somatic loss of *apt* expands the CySC population. **a-b** Projected images of optical sections of testes from males of the indicated genotypes, stained with antibodies directed against BrdU (magenta, to label dividing cells) and Tj (white). DAPI (blue) labels nuclei. The hub is outlined in blue. Scale bar = 20μm. Insets display single optical sections of indicated staining alone. **a** In control testes, CySCs divide at the hub (arrow). **b** When *apt* is reduced in the soma, a Tj+ cell divides several cell diameters away from the hub (arrowhead). **c** Percentage of testes, of the indicated genotypes, in which BrdU+ somatic cells were observed. Quantification was separated between CySCs (first tier of Tj+ cells around the hub, blue) and Tj+ cells more than one somatic cell tier away from the hub (red). Two-tailed Fisher’s exact tests were performed to analyze significance of the data, where *p<0.05 and **p<0.01. “n” indicates number of testes scored

Next, we wanted to determine if loss of *apt* yielded additional CySCs/CySC-like cells. While the additional Zfh-1+ cells in mutants often lacked Eya and could be CySCs, a more definitive marker of a stem cell is a periodic exit from quiescence. To assay cell division, we tested Bromodeoxyuridine (BrdU) incorporation, which occurs in dividing cells during S-phase. When *apt* was reduced in the soma, we found a significant increase in testes with at least one BrdU+ somatic (Tj+) cell several cell diameters away from the hub, relative to control testes (Fig. 4a-c). During eye development, Apt facilitates the transition from G1 to S-phase of the cell cycle [81]. However, we did not observe a significant difference in cell division of Tj + cells immediately surrounding the hub between *apt* loss of function and control testes (Fig. 4c). Thus, the expansion of the Zfh-1+ population when *apt* is reduced cannot be explained by an increased rate of CySC proliferation, and more likely indicates prolonged maintenance of CySC-like cells. Collectively, these data suggest Apt facilitates the differentiation program of CySCs.

### Apontic inhibits STAT activity in CySCs

**Fig. 5:**
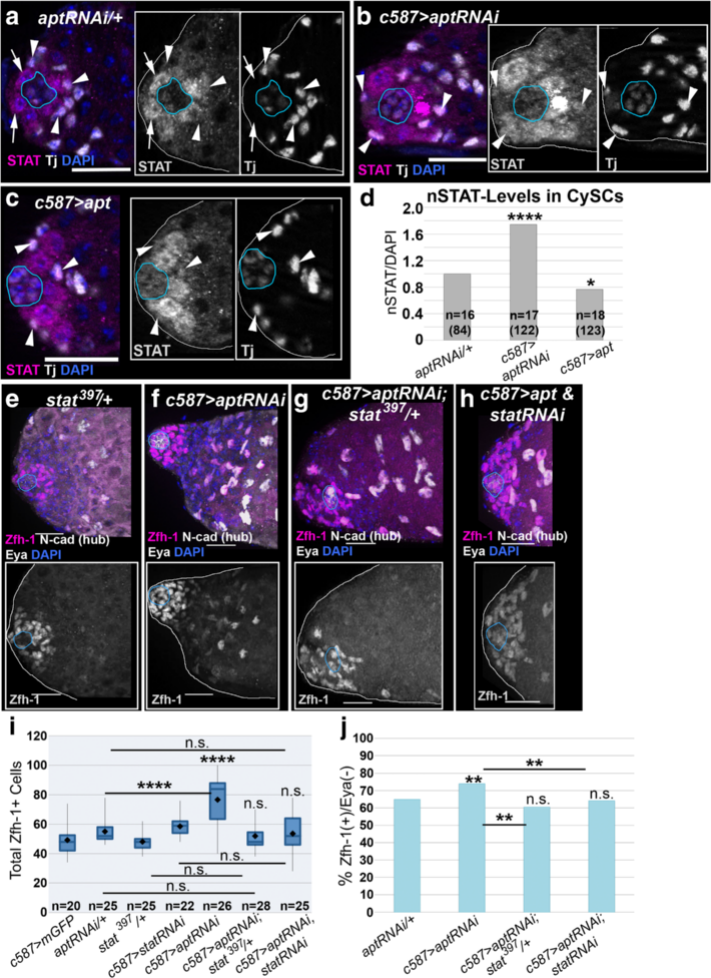
Apontic functions as a feedback inhibitor of STAT in the testes CySCs. **a-c** Testes stained with antibodies recognizing STAT (magenta) and Tj (white, somatic cells including the hub: blue outline) and counterstained with DAPI (blue, nuclei). Arrowheads indicate CySCs (first tier of Tj+ cells around the hub). Insets display STAT expression alone. **a** Control testis shows wild-type STAT expression: most detectable STAT is found in the GSCs around the hub (arrows), but it decreases in gonialblasts and CySCs (arrowheads). **b** More STAT is detectable when *apt* is reduced in somatic cells via *c587-Gal4*. **c** STAT expression is reduced in the CySCs when *apt* is over-expressed via *c587-Gal4*. **d** Nuclear STAT (nSTAT) levels were quantified in CySCs and normalized to DAPI intensity. Tj staining was utilized to outline nuclei of CySCs for measurement (see Methods). *c587-Gal4; aptRNAi* and *c587-Gal4;; UAS-apt* were normalized to the RNAi-alone control to obtain a relative expression level. Somatic reduction of *apt* significantly increases nSTAT levels in CySCs, while heightened levels of *apt *significantly reduces nSTAT in CySCs. **e-h** Optical sections projected into 2D of testes stained with Zfh-1 (magenta), Eya (white), and NCad (white) antibodies. Insets display Zfh-1 expression. Hub, indicated by NCad expression, is outlined in blue. A single copy mutation in *Stat92E* (**g**) or reduction of *Stat92E* via *c587-Gal4* (**h**) in testes with reduced *apt* in CySCs and early cyst cells suppresses the expansion of the Zfh-1+ population observed when *apt* alone is reduced (**f**) and is similar to the control (**e**). **i** Quantification of the Zfh-1+ population of indicated genotypes. Genetic reduction of *Stat92E* function (globally or in CySCs and early cyst cells) when *apt *is reduced via *c587-Gal4* results in a wild-type quantity of Zfh-1+ cells. **j** Proportion of Zfh-1+ cells that did not co-express the differentiation marker Eya in testes from the genotypes in (**i**). One copy of a *Stat92E* mutation in an *apt *deficient background restores the Zfh-1+/Eya- percentage to wild type. For all images scale bar = 20μm. Two-tailed t-tests were utilized to assess significance in (**d**) and (**i**), while a two-tailed Fisher’s exact test was used in (**j**), where *p<0.05, **p<0.01, ***p<0.005, ****p<0.0001, and n.s. denotes not significant. All statistics were performed between the experimental genotype and *aptRNAi*/+ (**d, j**) or *c587>mGFP* (**i**) controls unless otherwise indicated by a black bar. “n” indicates number of testes scored

The expanded Zfh-1+ population in *apt* mutants resembles the phenotype caused by excessive STAT signaling in the soma [23]. To determine if Apt inhibits STAT activity in testes, as it does in ovaries, we altered somatic *apt* expression. We expressed *apt* below and above endogenous levels via *aptRNAi* and a *UAS-apt* transgene, respectively, with *c587-Gal4.* We monitored nuclear STAT (nSTAT) protein levels in CySCs (the first tier of Tj + cells surrounding the hub), since this sub-cellular localization provides a read-out for STAT activity [64]. Somatic reduction of *apt* cell-autonomously increased nSTAT protein levels relative to the control (compare arrowheads in Fig. 5a and b). Conversely, high levels of *apt* in the CySC and early cyst cell populations resulted in decreased nSTAT in CySCs (Fig. 5c). Quantification of this expression pattern revealed that the detectable amounts of nSTAT in CySCs changed significantly when *apt* was reduced or heightened (Fig. 5d and a-c, arrowheads). We obtained similar results using the *Tj-Gal4* driver (Additional file 4: Figure S4A-C). Interestingly, we observed Tj+, nSTAT+ cells several cell diameters away from the hub when *apt* was reduced (Additional file 4: Figure S4B asterisks), consistent with an expanded Zfh-1+ (CySC-like) population. We did not observe this expression pattern in control testes (Additional file 4: Figure S4A). These results support the idea that Apt negatively regulates STAT activity.

If Apt attenuates STAT signaling, then the *apt* mutant phenotype should be suppressed by reducing *Stat92E* expression. In support of this, when *apt* expression was lowered, we found that additional removal of one functional copy of the *Stat92E* gene*,* or reducing *Stat92E* expression in CySCs and early cyst cells using *c587-Gal4,* resulted in a Zfh-1+ population that was approximately wild type in cell numbers and organization (Fig. 5e-i). Reduction of *Stat92E* function, globally or via *c587-Gal4*, lowered the percentage of Zfh-1+, Eya(-) cells to wild type (Fig. 5j). Given the expression pattern and mutant analysis, we propose that Apt functions as a feedback inhibitor of STAT signaling in adult CySCs, which enables a proper spatiotemporal transition from CySC to mature cyst cell.

### Apt functions in a STAT-regulatory genetic circuit to promote cyst cell development

**Fig. 6:**
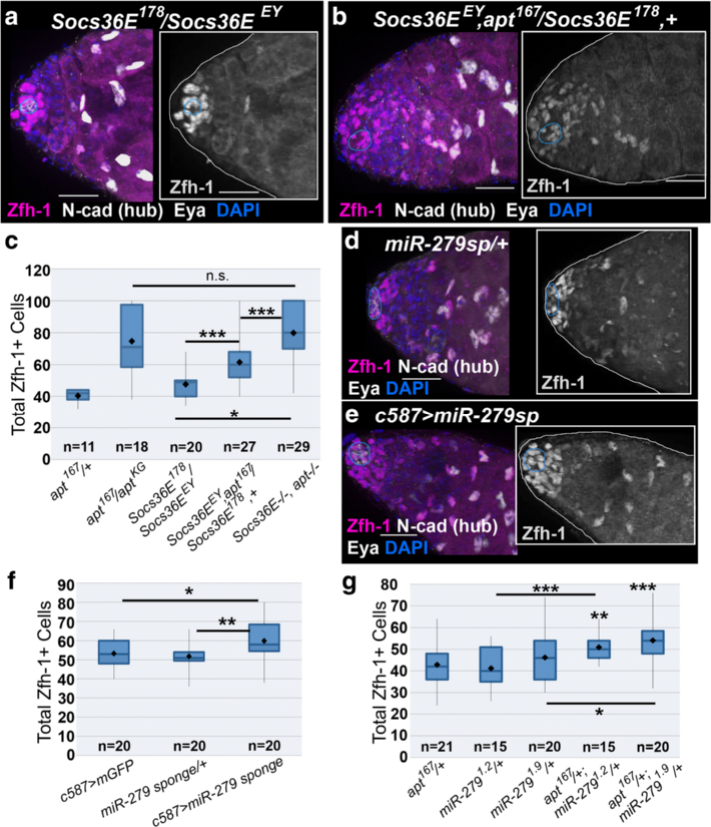
Apontic inhibits STAT function via a conserved genetic circuit in testes. **a-b, d-e** 2-D projections of optical sections of testes stained with antibodies directed against Zfh-1 (magenta), Eya (white), and N-Cad (white, to label hub, outlined in blue). Insets show Zfh-1 expression alone. **a** A testis from a *Socs36E* mutant male contains a wild-type number and organization of Zfh-1+ cells. **b** A single copy reduction of *apt* function (via null allele *apt* ^*167*^) in a *Socs36E* ^*EY06665*^/*Socs36E* ^*178*^ mutant male significantly expands the Zfh-1+ population in the testis. **c** Zfh-1+ cell quantification in the specified genotypes shows a genetic interaction between *apt* and *Socs36E*. **d** Testis from a male bearing the *UAS-miR-279sponge* (*miR-279sp*) with no Gal4 driver displays a wild-type number and arrangement of Zfh-1+ cells. **e** The Zfh-1+ population is expanded when the *miR-279sponge* is expressed via *c587-Gal4*. **f** Total Zfh-1+ cell number increases when *miR-279sponge* is expressed. **g** Quantification of Zfh-1+ cells in the specified genotypes reveals a genetic interaction between *apt* and *miR-279*. For all images the scale bar = 20 μm. For graphs, two-tailed t-tests were used to assess significance where n.s. denotes not significant, *p<0.05, ***p<0.005, ****p<0.0001. Significance was measured between the experimental genotype(s) and *apt* ^*167*^/+ (**c, g**) or *c587>GFP* (**f**) unless otherwise indicated by a bar. “n” indicates number of testes scored

Apt activates expression of STAT inhibitors during oogenesis [41, 47, 48]. To determine if Apt has similar targets in testes, we examined *Suppressor of cytokine signaling at 36E* (*Socs36E*) – a conserved post-transcriptional regulator of STAT [77, 82]. Socs36E inhibits STAT activity in CySCs and regulates their adhesion to the hub [31, 83]; however, loss of *Socs36E* does not increase the Zfh-1+ population (Fig. 6a, c and [31]). Using the hypomorphic allele *Socs36E*^*EY06665*^ and the strong loss of function allele *Socs36E*^*178*^ [47], we looked for a genetic interaction between *Socs36E* and *apt. Socs36E*, *apt* double homozygous mutants displayed an expanded Zfh-1+ population very similar to loss of *apt* alone (Fig. 6c). Interestingly, reduction of *apt* in a *Socs36E* deficient background (*Socs36E*^*EY06665*^*, apt*^*167*^*/Socs36E*^*178*^*, +*) partially phenocopied the Zfh-1+ cell number expansion observed in testes from *apt* homozygous mutant males (Fig. 6b (compare with Fig. 3c), c, and [31]). These results are consistent with the idea that Apt functions through *Socs36E* in CySCs.

Apt promotes expression of the STAT-targeting microRNA *miR-279* in ovaries [48]. To test if this regulator also acts in CySCs, we utilized a *miR-279 sponge*, which binds and decreases the endogenous microRNA [48, 84]. Expressing the *miR-279 sponge* in CySCs and early cyst cells via *c587-Gal4* led to a significant increase in the total number of Zfh-1+ cells (Fig. 6d-f). To determine if *miR-279* function depends on or overlaps with Apt in testes, we generated *apt-/+; miR-279-/+* double heterozygous flies [56]. Testes from these flies had significantly more Zfh-1+ cells relative to a single copy reduction of *miR-279* or *apt* alone (Fig. 6g). Combined, these data suggest that somatic STAT activity is finely tuned via a genetic regulatory circuit that is conserved in distinct processes during male and female gametogenesis.

### Apt limits the GSC population at the hub interface

While the hub is a key organizer of the niche, CySCs also function as a GSC niche component [23, 24, 85–87]. Since loss of *apt* function results in an expanded Zfh-1+ population, we examined whether the GSCs were affected. Using an antibody for Vasa to label germ cells [65], we assessed the GSC population when *apt* was reduced in the soma by RNAi, focusing near the niche. For this, we defined a GSC as a Vasa-positive single cell directly interacting with the hub. Despite an increase in Zfh-1+ cells, we found no change in the number of GSCs at the hub interface when *apt* was reduced in CySCs and early cyst cells (*c587 > aptRNAi*) relative to the GFP control (9.6+/-2.0 GSCs on average, versus 9.8+/-2.8, respectively: *p* > 0.05).

In contrast, examination of the GSC population in testes of males homozygous for the *apt*^*KG05830*^ allele or bearing two different *apt* alleles (*apt*^*KG05830*^*/apt*^*tdf-P*Δ*4*^) [41, 53] revealed a significant increase in the number of GSCs, relative to control testes (an average of 13.2+/-2.3 GSCs per testis in *apt*^*KG05830*^ homozygotes, 12.1+/-1.8 in *apt*^*KG05830*^*/apt*^*tdf-P*Δ*4*^ heteroallelic mutants, and 9.0+/-2.5 in Canton S: *p* < 0.0001 for either mutant genotype compared to Canton S, and Additional file 5: Figure S5A-C). These data suggest Apt may function in the GSCs.

STAT regulates E-cad in the germline [24]. Consistent with Apt functioning as a STAT-regulator in the stem cell populations, we found altered expression of E-cad in the cell populations directly surrounding the hub in *apt*^*KG05830*^ homozygous males, relative to wild-type controls (compare Additional file 5: Figure S5E with D). Specifically, we detected E-cad all around the cells, not just at the hub interface, suggesting it was more highly expressed, or mislocalized, or both. Co-staining with an antibody targeting Vasa verified that GSCs were among the cells with heightened E-cad accumulation.

Since allelic and tissue-specific reduction of *apt* both resulted in an expanded Zfh-1+ population (Fig. 3a and e), we set out to determine why we observed distinct GSC phenotypes. Assessment of *apt*^*KG05830*^ homozygotes by antibody staining revealed that Apt protein is mildly reduced in CySCs but is expressed below detectable levels in GSCs (Additional file 5: Figure S5F-H). Collectively, these data support the possibility that significant loss of *apt* in the GSCs, relative to CySCs, alters GSC adhesion to the hub resulting in their accumulation. Thus, these data may suggest Apt is required in both stem cell types, and that the levels of *apt* expression must be fine-tuned between each population to establish a balance at the hub interface.

## Discussion

Here, we show that the transcriptional regulator Apt is required for maintaining a wild-type number of stem cells in the testis apex. Within CySCs, this function is dependent on Apt’s inhibition of STAT activity, which promotes stem cell differentiation cell-autonomously. We found that a high level of conservation exists in a STAT-feedback genetic regulatory network that limits this stem cell population as well as cell invasion in ovarian follicle cells [41, 47, 48]. Despite obvious distinctions between these processes, each requires a correct number of different cell types to be allocated to the tissue. Pathways essential for each process, such as JAK/STAT, are often aberrantly activated in pathogenesis, such as cancer development, and thus require intense regulation [12, 36, 88].

### A conserved genetic regulatory circuit is essential for stem cell differentiation

Apt is expressed in the adult testes somatic population (hub, CySCs, and cyst cells) and GSCs and gonialblasts. We verified that *apt* is expressed downstream of STAT in the CySCs. However, mature cyst cells do not have detectable STAT activation, which suggests that, like in the egg chamber, *apt* is regulated in STAT-dependent and independent manners [41, 42]. While Apt is expressed in all anterior follicle cells of the egg chamber, STAT signaling in the anterior epithelium is more restricted [41, 45–47]. Eya, which is expressed in a similar pattern as Apt in follicle cells, is required to activate *apt* expression broadly [41, 42]. In wild-type somatic cells of the testes, STAT activity and Eya expression have complementary patterns – Eya is not expressed until cyst cell maturation, when STAT is turned off [15, 80]. Thus, in CySCs and early cyst cells Apt is regulated by STAT, then akin to the ovary, Eya may maintain Apt expression in the mature cyst cells.

**Fig. 7:**
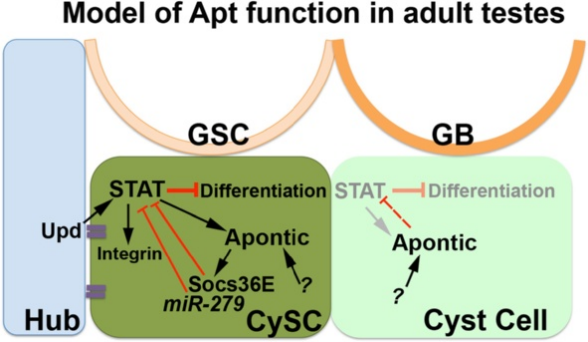
Apt functions as a feedback inhibitor of the JAK/STAT pathway in CySCs. Apontic (Apt) is required in the CySCs as a feedback inhibitor of STAT signaling to promote the CySC to cyst cell transition: *see text for details.* Apt’s genetic interactions with the STAT-targeting *miR-279* and the conserved inhibitor of STAT, *Socs36E*, suggest it may mediate expression of these targets, as in ovaries. GSC=Germline Stem Cell, CySC=Cyst Stem Cell

We found that in the Drosophila testis Apt is a keystone in a STAT genetic regulatory network, acting as a feedback inhibitor in somatic stem cells (Fig. 7). Of the three previously described STAT downstream targets in CySCs – *zfh-1, chinmo,* and *Socs36E* – two prevent CySC differentiation [21, 23], while Socs36E affects CySC adhesion, but not fate [31]. Our data suggest *apt* is distinct in that it permits the transition from CySC-to-mature cyst cell. While self-renewal of stem cell populations is essential, the ability to differentiate is also critical for tissue repair and regeneration [1, 2, 8]. For differentiation to occur in CySCs, STAT signaling must be shut down in the daughter dividing away from the hub [19, 23]. Loss of *apt* in CySCs delays this transition, reflected in a significant expansion of the Zfh-1+ (and Eya(-)) population that maintains the potential to undergo cell division away from the hub. While clonal mutant analysis using a null allele of *apt* in the CySCs might help clarify Apt’s distinct roles, we have been unsuccessful in obtaining males of the necessary genotype for this experiment, despite multiple attempts. Nevertheless, the results from viable mutants as well as temperature-regulated knockdown in overlapping expression domains consistently and strongly suggest Apt is a feedback inhibitor of the JAK/STAT pathway that is required for CySC maturation.

STAT regulates CySC adhesion to the hub [31]. It is, therefore, conceivable to think that the expansion of the Zfh-1+ population in *apt* mutants is partially due to increased adhesion of the CySCs and early cyst cells to the niche area. This would enable these cells to continue to receive the self-renewal cues. Furthermore, the amount of CySC division did not appear altered between control and loss of *apt* testes, which suggests that Apt does not work through *Cyclin E* regulation in this tissue [81]. Instead, we discovered a significant increase in nuclear STAT levels in CySCs with reduced *apt*. Thus, Apt is required in CySCs to attenuate STAT, which allows efficient and correct spatiotemporal somatic cell maturation in adults.

Loss of the STAT feedback inhibitor *Socs36E* increases STAT activity without affecting CySC numbers [31]. However, when we removed a single copy of *apt* in a *Socs36E* mutant background, the Zfh-1+ population significantly increased. Although Apt can activate *Socs36E* expression [47], the inability of *Socs36E* reduction to alter the number of CySCs led us to hypothesize Apt may inhibit STAT through another regulator, as well. A genetic interaction between *apt* and the *Stat92E* targeting *miR-279* [48] supports this idea. Unlike loss of *Socs36E*, reduction of *miR-279* in the CySC and early cyst cell populations significantly expanded the Zfh-1+ population. While we cannot rule out any direct regulation of STAT by Apt, our data strongly suggest a conserved genetic circuit attenuates STAT activity in CySCs, which is required to allocate an appropriate balance of cells that will self-renew and differentiate (Fig. 7 and [47, 48]).

Similarly when *apt* was lost in the germline, we observed a significant accumulation of GSCs at the hub interface. STAT has been shown to promote the expression of E-cad in GSCs [24], and integrin expression in CySCs [31–33], in both cases facilitating interaction with the hub. Loss of Apt in the germline resulted in heightened E-cad expression in the GSCs. These data support a model in which Apt negatively regulates STAT activity in the GSCs and that the loss of *apt* may increase the adhesiveness of GSCs to the hub. The mild reduction of Apt expression in the CySCs in these experiments may suggest that the relative levels of *apt* between the two stem cell populations mediates a balance between the stem cell populations - possibly through adhesion molecule expression, since adherence to the hub is important for maintaining stem-ness in both cell types. Collectively, our data suggest *apt* is essential in both stem cell populations of the Drosophila testes to ensure appropriate maintenance and differentiation.

## Conclusions

In summary, we postulate that Apt functions as a feedback inhibitor of JAK/STAT activation in the CySCs via its regulation of *Socs36E* and *miR-279* (Fig. 7). This genetic regulatory network is essential to prevent stem cell over-population by enabling CySC maturation. Our data also suggest that appropriate expression levels of *apt* between the CySCs and GSCs are important to maintain a balance of the stem cell populations at the hub interface. Collectively, this work shows that Apt is an important regulator of stem cell dynamics in the Drosophila testis.

## Additional files

Additional file 1: Figure S1. Apt is expressed in the somatic population and GSCs. Single optical section through the apex of a testis from a *PTT-apt* male stained with antibodies specific for Apt (magenta), E-cadherin (green), and GFP as a read out for Apt expression (green, PTT-Apt). DAPI (blue) labels nuclei. A similar expression pattern for Apt is observed with an antibody targeting Apt and the protein trap for Apt (Apt-GFP): Apt is expressed in the hub (outlined in blue), CySCs (arrowhead), and GSCs (arrow). E-cadherin expression is also shown with Apt-GFP. For comparison, wild-type E-cad expression alone is shown in Additional file 5: Figure S5D. Insets show anti-Apt or Apt-GFP staining alone. Pattern of expression is similar to that observed in Figures 1c-d. (TIFF 874 kb) Additional file 2: Figure S2. Reduction of *apt* results in an increase of somatic cells in the testis and excessive cell migration in the ovary. **A**-**B**) Single optical sections of testes from males of specified genotypes stained with antibodies specific for the germline marker Vasa (magenta), the somatic marker Tj (green), and the hub marker Fas3 (green). DAPI (blue) labels DNA. Insets display stacks of optical sections of the Tj and Fas3 expression projected into 2D; a similar tissue depth was used to generate the projections for control and experimental testes. Dashed box indicates the region of the testis that is shown in the single optical section to the left. The hub is outlined in blue. Scale bars = 20 μm. **A**) In control testes (*apt* ^*167*^/+), wild - type arrangement and numbers of somatic cells are observed: Tj + cells are adjacent to a germline cell. **B**) An expansion of the somatic population is observed when *apt* is lost: arrowhead indicates an accumulation of Tj + cells at the testis apex. **C**-**D**) Optical section images of egg chambers stained with antibodies specific for GFP or E-cadherin (green, labels anterior follicle cells and border cells or the whole epithelium, respectively) and Eya (red, labels all anterior follicle cells, except polar cells). DNA is visualized with DAPI. Anterior is to the left, scale bars = 50 μm. An anterior follicle cell (AFC) driver, *c306-Gal4*, is expressed in the border cell population during their specification (stage 8) through migration (stages 9-10). **C**) Control stage 9 egg chamber displays normal border cell specification (invasion is limited to the cluster: arrow) and migration. **D**) Stage 10 egg chamber: *c306-Gal4; aptRNAi* results in excessive invasive follicle cells (arrowheads), a phenotype that mimics the prior descriptions of *apt* loss of function and shows that the *aptRNAi* line has on-target effects. Arrow indicates the border cell cluster at the oocyte. (TIFF 4523 kb) Additional file 3: Figure S3. Apontic limits the Zfh-1+ cell population. Single optical sections (**A**, **C**, **E**) or 2D projections of stacks of optical sections (**B**, **D**, **F**, **G**) of testes from males of indicated genotypes and stained with antibodies specific for Zfh-1 (magenta), Eya (white), and N-cad (white, to label the hub, outlined in blue). Insets show Zfh-1 expression alone. **A**-**B**) A control testis expressing membrane GFP (mGFP; white) in the cyst stem cells and early cyst cells shows a wild-type number and arrangement of Zfh-1+ cells. **C**) A testis with *apt* expression reduced in CySCs and early cyst cells via *c587-Gal4* contains an expanded Zfh-1+ population. Despite the excess Zfh-1+ cells, GSCs remain present at the hub interface (arrow). **D**) A projected image from this genotype shows more Zfh-1+ cells at the hub and a smaller number distally. **E**-**F**) Re-introduction of *apt* in an RNAi background results in a wild-type number and organization of the Zfh-1+ cell population. **G**) Fewer Zfh-1+ cells are observed in a testis with above endogenous levels of *apt* in the CySC and early cyst cell populations. In this genotype, Zfh-1+ cells remain adjacent to the hub. Scale bars = 20 μm for all images. **H**) Expression of *aptRNAi* in the soma via *Tj-Gal4* significantly expands the Zfh-1+ population, as with *c587-Gal4* (see Fig. 3f). Ectopic levels of *apt* in the soma reduces the number of Zfh-1+ cells. Two-tailed t-tests were utilized for significant analysis, where **p* < 0.05 and ****p* < 0.005. (TIF 8293 kb) Additional file 4: Figure S4. Somatic reduction of *apt* heightens STAT expression in the CySCs. **A-B**) Testes stained with antibodies recognizing STAT (magenta), Tj (white, somatic cells), and Fas3 (white, hub: blue outline) and counterstained with DAPI (blue, nuclei). Arrowheads indicate CySCs (first tier of Tj + cells around the hub). Scale bars = 20 μm. Insets display STAT expression, alone. **A**) Control testis shows wild - type STAT expression: most detectable STAT is found in the GSCs around the hub (labeled arrows), but it decreases in gonialblasts and CySCs (arrowheads). A Tj + cell distal from the hub shows undetectable levels of nSTAT (unlabeled arrow). **B**) More STAT is detectable when *apt* is reduced in somatic cells via *Tj-Gal4*. Tj + cells several cell diameters away from the hub displayed high levels of nSTAT (asterisks). **C**) Nuclear STAT (nSTAT) levels were quantified in CySCs and normalized to DAPI intensity. Tj staining was utilized to outline nuclei of CySCs for measurement (see Methods). *Tj-Gal4*;*aptRNAi* was normalized to the *Tj-Gal4* or *aptRNAi*-alone controls to obtain a relative expression level. Somatic reduction of *apt* significantly increases nSTAT levels in CySCs. Two-tailed t-tests were used to test for significance, as indicated. “n” provides the total number of testes examined for each genotype, while the number of individual cells analyzed is given in parentheses. (TIF 8047 kb) Additional file 5: Figure S5. Apt limits the GSC population at the hub interface and E-cadherin expression. **A**-**B**) Single optical sections of testes stained with antibodies that recognize Vasa (magenta), Tj (white), and Fas3 (white, to label the hub). The hub is outlined in blue. Scale bars = 10 μm. Testes from homozygous *apt* ^*KG05830*^ males exhibit a significant increase in GSCs (magenta) contacting the hub (**B**), relative to wild type (**A**). **C**) Number of GSCs at the hub interface for the indicated genotypes. **D**-**E**) Images of testes stained with antibodies specific for E-cadherin (magenta and insets), Vasa (white), and DAPI (blue). Scale bars = 20 μm. An increase or mislocalization of E-cadherin expression is observed in the cells surrounding the hub, including the Vasa + GSCs (arrows) in a testis from an *apt* ^*KG05830*^ homozygous male (**E**), compared to a *w* ^*1118*^ testis (**D**, arrows), where it is barely detected outside the hub. Images were taken under the same conditions. **F**-**G**) Single optical sections of testes stained for Apt (magenta and insets), Tj (white), and DAPI (blue). Arrows indicate GSCs; arrowheads show CySCs. Scale bars = 10 μm. **F**) *apt* ^*KG05830*^ /+ heterozygotes show no significant reduction of Apt protein in CySCs and a mild reduction in GSCs. **G**) In homozygous mutant males, Apt expression is reduced in CySCs (first tier of Apt+/Tj + cells proximal to the hub: arrowheads) but is not detected in the germline (arrows, the presence of a cell is indicated by DAPI). **H**) Quantification of the relative expression levels of Apt protein in the stem cell populations adjacent to the hub for the indicated genotypes. "n" is the number of testes examined with the number of cells in parentheses. Statistical significance was tested via two-tailed t-tests, where **p* < 0.05, ****p* < 0.005, *****p* < 0.0001, and n.s. = not significant. Experimental genotypes were tested against Canton S, unless indicated by a bar. (TIF 14942 kb)

## Abbreviations

Apt: Apontic
CySC: Cyst stem cell
E-cad: E-cadherin
Eya: Eyes absent
GSC: Germline stem cell
JAK/STAT: Janus Kinase/Signal Transducer and Activator of Transcription
Socs36E: Suppressor of cytokine signaling at 36E
Zfh-1: Zinc finger homeodomain-1
