# Supplementary figures and images for: Apontic regulates somatic stem cell numbers in Drosophila testes
Source: BMC Dev Biol. 2016 Mar 18;16:5. doi: 10.1186/s12861-016-0103-3 (PMC4799534; doi:10.1186/s12861-016-0103-3)

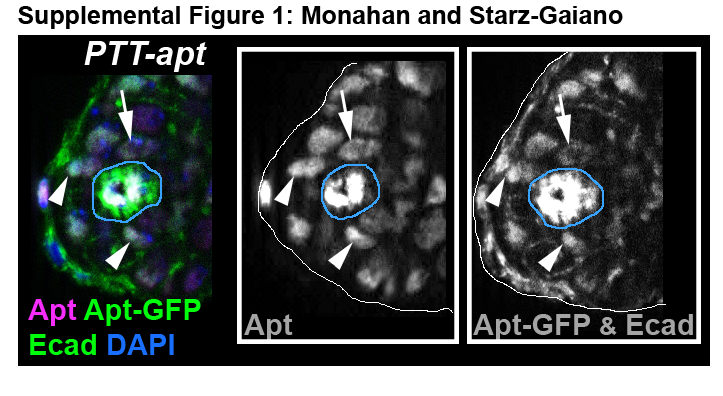

Supplement: Additional file 1: Figure S1. — Apt is expressed in the somatic population and GSCs. Single optical section through the apex of a testis from a PTT-apt male stained with antibodies specific for Apt (magenta), E-cadherin (green), and GFP as a read out for Apt expression (green, PTT-Apt). DAPI (blue) labels nuclei. A similar expression pattern for Apt is observed with an antibody targeting Apt and the protein trap for Apt (Apt-GFP): Apt is expressed in the hub (outlined in blue), CySCs (arrowhead), and GSCs (arrow). E-cadherin expression is also shown with Apt-GFP. For comparison, wild-type E-cad expression alone is shown in Additional file 5: Figure S5D. Insets show anti-Apt or Apt-GFP staining alone. Pattern of expression is similar to that observed in Figures 1c-d. (TIFF 874 kb) [file 12861_2016_103_MOESM1_ESM.tiff]

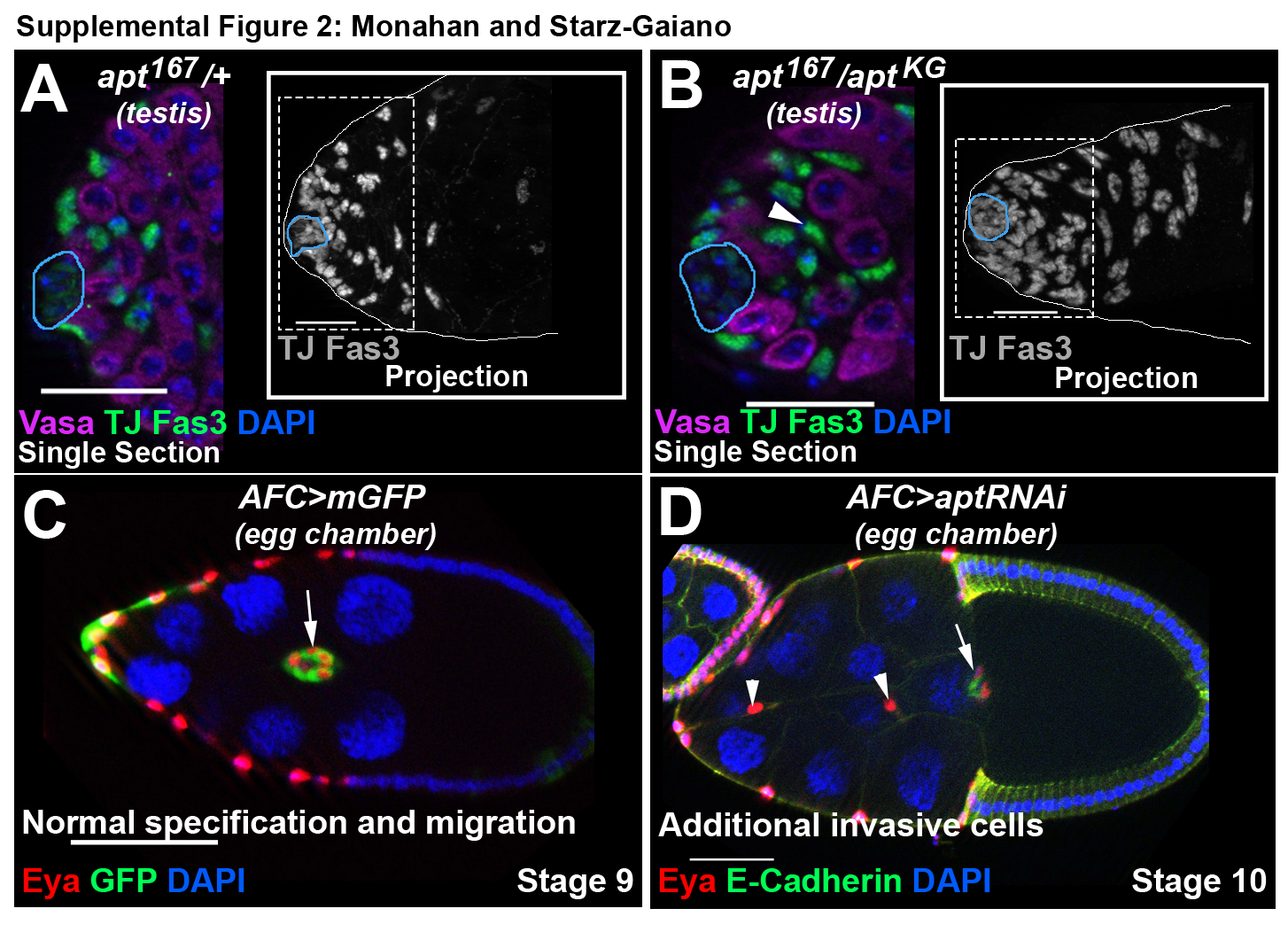

Supplement: Additional file 2: Figure S2. — Reduction of apt results in an increase of somatic cells in the testis and excessive cell migration in the ovary. A-B) Single optical sections of testes from males of specified genotypes stained with antibodies specific for the germline marker Vasa (magenta), the somatic marker Tj (green), and the hub marker Fas3 (green). DAPI (blue) labels DNA. Insets display stacks of optical sections of the Tj and Fas3 expression projected into 2D; a similar tissue depth was used to generate the projections for control and experimental testes. Dashed box indicates the region of the testis that is shown in the single optical section to the left. The hub is outlined in blue. Scale bars = 20 μm. A) In control testes (apt 167/+), wild - type arrangement and numbers of somatic cells are observed: Tj + cells are adjacent to a germline cell. B) An expansion of the somatic population is observed when apt is lost: arrowhead indicates an accumulation of Tj + cells at the testis apex. C-D) Optical section images of egg chambers stained with antibodies specific for GFP or E-cadherin (green, labels anterior follicle cells and border cells or the whole epithelium, respectively) and Eya (red, labels all anterior follicle cells, except polar cells). DNA is visualized with DAPI. Anterior is to the left, scale bars = 50 μm. An anterior follicle cell (AFC) driver, c306-Gal4, is expressed in the border cell population during their specification (stage 8) through migration (stages 9-10). C) Control stage 9 egg chamber displays normal border cell specification (invasion is limited to the cluster: arrow) and migration. D) Stage 10 egg chamber: c306-Gal4; aptRNAi results in excessive invasive follicle cells (arrowheads), a phenotype that mimics the prior descriptions of apt loss of function and shows that the aptRNAi line has on-target effects. Arrow indicates the border cell cluster at the oocyte. (TIFF 4523 kb) [file 12861_2016_103_MOESM2_ESM.tiff]

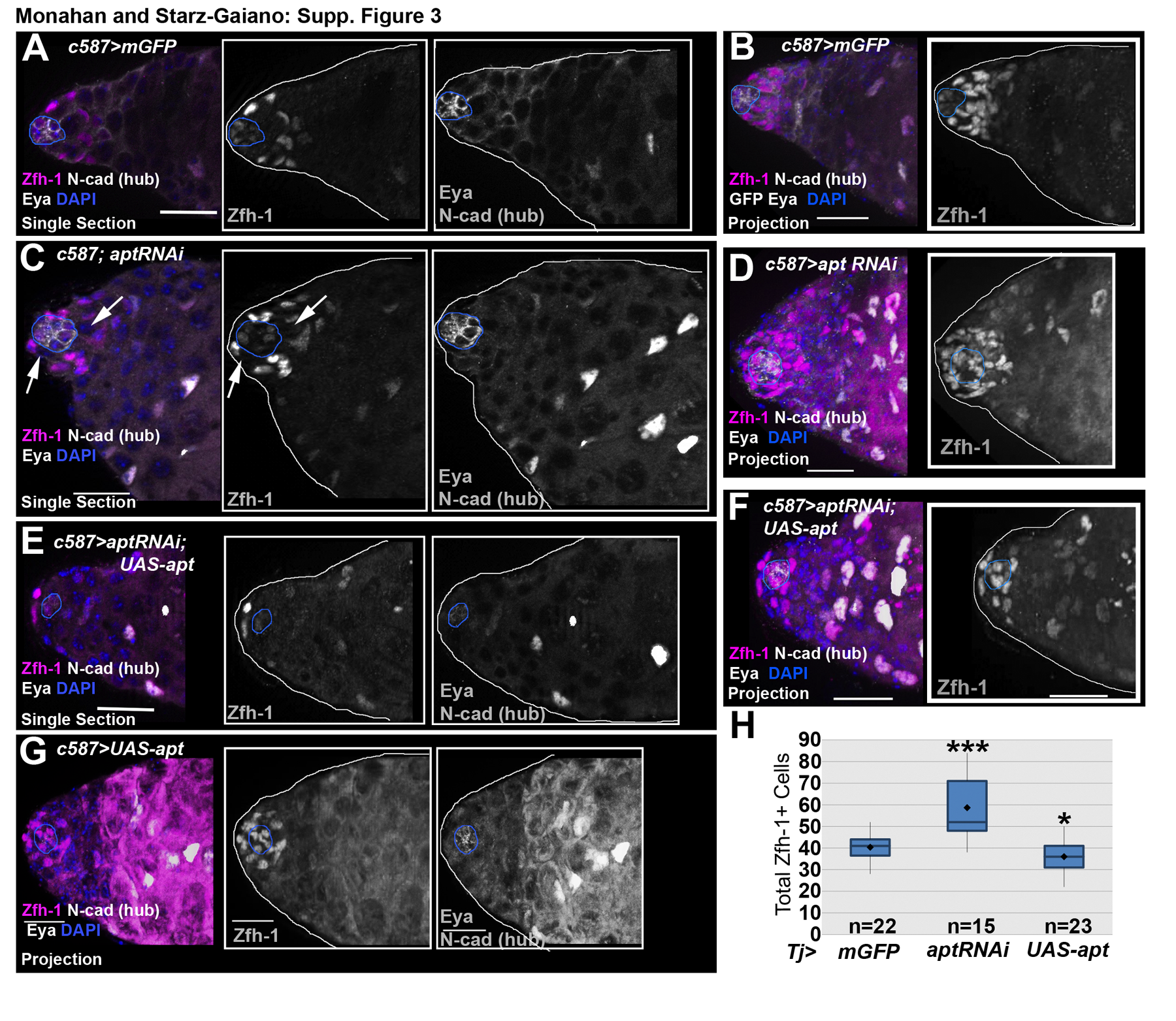

Supplement: Additional file 3: Figure S3. — Apontic limits the Zfh-1+ cell population. Single optical sections (A, C, E) or 2D projections of stacks of optical sections (B, D, F, G) of testes from males of indicated genotypes and stained with antibodies specific for Zfh-1 (magenta), Eya (white), and N-cad (white, to label the hub, outlined in blue). Insets show Zfh-1 expression alone. A-B) A control testis expressing membrane GFP (mGFP; white) in the cyst stem cells and early cyst cells shows a wild-type number and arrangement of Zfh-1+ cells. C) A testis with apt expression reduced in CySCs and early cyst cells via c587-Gal4 contains an expanded Zfh-1+ population. Despite the excess Zfh-1+ cells, GSCs remain present at the hub interface (arrow). D) A projected image from this genotype shows more Zfh-1+ cells at the hub and a smaller number distally. E-F) Re-introduction of apt in an RNAi background results in a wild-type number and organization of the Zfh-1+ cell population. G) Fewer Zfh-1+ cells are observed in a testis with above endogenous levels of apt in the CySC and early cyst cell populations. In this genotype, Zfh-1+ cells remain adjacent to the hub. Scale bars = 20 μm for all images. H) Expression of aptRNAi in the soma via Tj-Gal4 significantly expands the Zfh-1+ population, as with c587-Gal4 (see Fig. 3f). Ectopic levels of apt in the soma reduces the number of Zfh-1+ cells. Two-tailed t-tests were utilized for significant analysis, where *p < 0.05 and ***p < 0.005. (TIF 8293 kb) [file 12861_2016_103_MOESM3_ESM.tif]

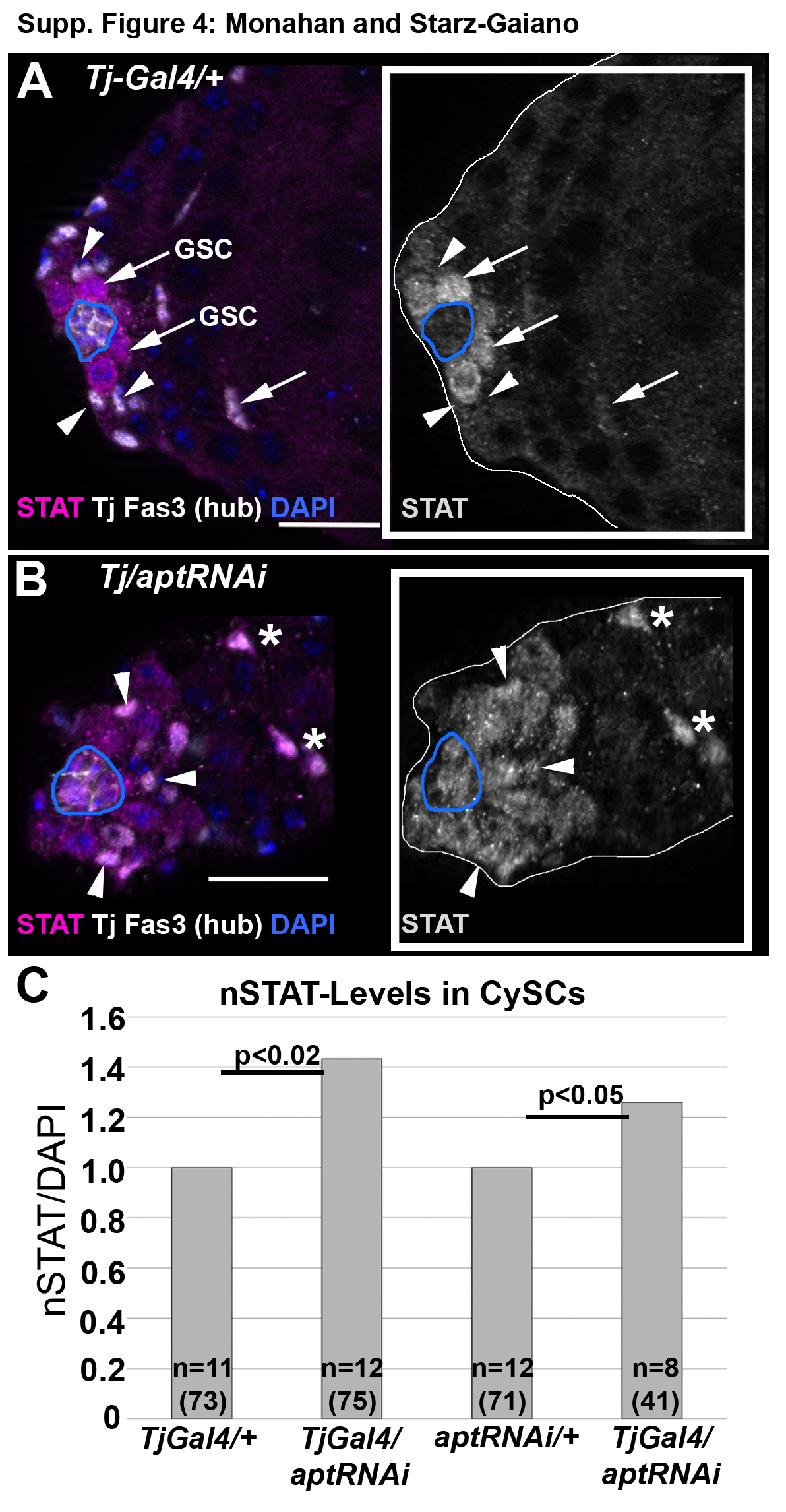

Supplement: Additional file 4: Figure S4. — Somatic reduction of apt heightens STAT expression in the CySCs. A-B) Testes stained with antibodies recognizing STAT (magenta), Tj (white, somatic cells), and Fas3 (white, hub: blue outline) and counterstained with DAPI (blue, nuclei). Arrowheads indicate CySCs (first tier of Tj + cells around the hub). Scale bars = 20 μm. Insets display STAT expression, alone. A) Control testis shows wild - type STAT expression: most detectable STAT is found in the GSCs around the hub (labeled arrows), but it decreases in gonialblasts and CySCs (arrowheads). A Tj + cell distal from the hub shows undetectable levels of nSTAT (unlabeled arrow). B) More STAT is detectable when apt is reduced in somatic cells via Tj-Gal4. Tj + cells several cell diameters away from the hub displayed high levels of nSTAT (asterisks). C) Nuclear STAT (nSTAT) levels were quantified in CySCs and normalized to DAPI intensity. Tj staining was utilized to outline nuclei of CySCs for measurement (see Methods). Tj-Gal4;aptRNAi was normalized to the Tj-Gal4 or aptRNAi-alone controls to obtain a relative expression level. Somatic reduction of apt significantly increases nSTAT levels in CySCs. Two-tailed t-tests were used to test for significance, as indicated. “n” provides the total number of testes examined for each genotype, while the number of individual cells analyzed is given in parentheses. (TIF 8047 kb) [file 12861_2016_103_MOESM4_ESM.tif]

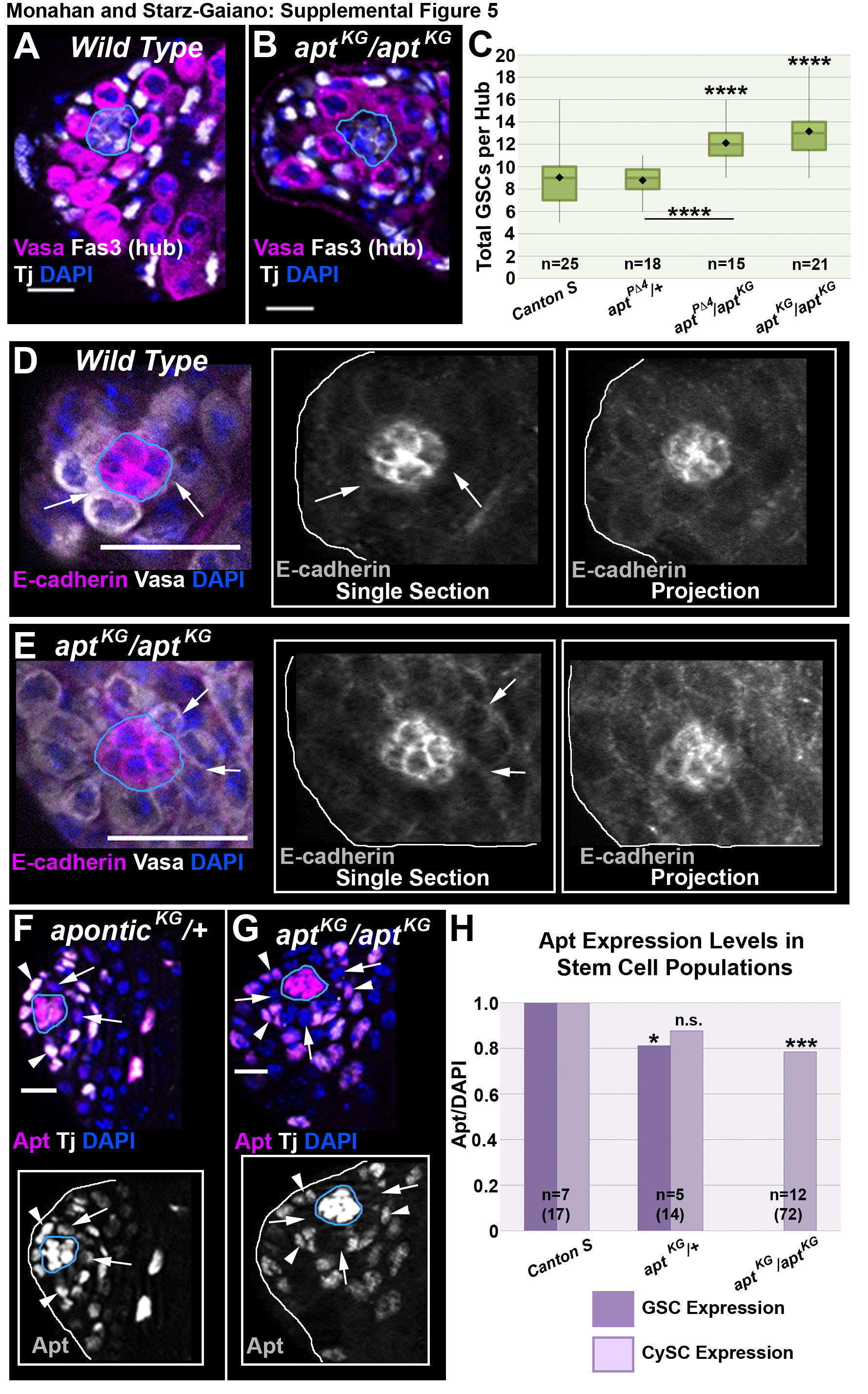

Supplement: Additional file 5: Figure S5. — Apt limits the GSC population at the hub interface and E-cadherin expression. A-B) Single optical sections of testes stained with antibodies that recognize Vasa (magenta), Tj (white), and Fas3 (white, to label the hub). The hub is outlined in blue. Scale bars = 10 μm. Testes from homozygous apt KG05830 males exhibit a significant increase in GSCs (magenta) contacting the hub (B), relative to wild type (A). C) Number of GSCs at the hub interface for the indicated genotypes. D-E) Images of testes stained with antibodies specific for E-cadherin (magenta and insets), Vasa (white), and DAPI (blue). Scale bars = 20 μm. An increase or mislocalization of E-cadherin expression is observed in the cells surrounding the hub, including the Vasa + GSCs (arrows) in a testis from an apt KG05830 homozygous male (E), compared to a w 1118 testis (D, arrows), where it is barely detected outside the hub. Images were taken under the same conditions. F-G) Single optical sections of testes stained for Apt (magenta and insets), Tj (white), and DAPI (blue). Arrows indicate GSCs; arrowheads show CySCs. Scale bars = 10 μm. F) apt KG05830 /+ heterozygotes show no significant reduction of Apt protein in CySCs and a mild reduction in GSCs. G) In homozygous mutant males, Apt expression is reduced in CySCs (first tier of Apt+/Tj + cells proximal to the hub: arrowheads) but is not detected in the germline (arrows, the presence of a cell is indicated by DAPI). H) Quantification of the relative expression levels of Apt protein in the stem cell populations adjacent to the hub for the indicated genotypes. "n" is the number of testes examined with the number of cells in parentheses. Statistical significance was tested via two-tailed t-tests, where *p < 0.05, ***p < 0.005, ****p < 0.0001, and n.s. = not significant. Experimental genotypes were tested against Canton S, unless indicated by a bar. (TIF 14942 kb) [file 12861_2016_103_MOESM5_ESM.tif]
